# Nutraceutical formulation based on a synergic combination of melatonin and palmitoylethanolamide for the management of allergic events

**DOI:** 10.3389/fnut.2024.1417747

**Published:** 2024-08-27

**Authors:** Maria Maisto, Vincenzo Piccolo, Adua Marzocchi, Daniela Claudia Maresca, Benedetta Romano, Vincenzo Summa, Gian Carlo Tenore, Giuseppe Ercolano, Angela Ianaro

**Affiliations:** ^1^ChimNutra Labs, Department of Pharmacy, University of Naples Federico II, Naples, Italy; ^2^Department of Pharmacy, University of Naples Federico II, Naples, Italy

**Keywords:** melatonin, palmitoylethanolamide, synergic combination, nutraceutical formulation, immune response, human mast cell

## Abstract

The management of allergic events is a growing global health issue, especially in industrialized countries. This disease is an immune-mediated process, regulated by the interaction of IgE with an allergen, resulting in mast cell activation, which concerns the release of several immune-inflammatory modulators, i.e., histamine, β-hexosaminidase, COX-2, IL-6, and TNF-α, responsible for the main allergic-reaction associated symptoms. The aim of the present study was the efficacy evaluation of an alternative remedy, an innovative nutraceutical formulation (NF) based on the synergic combination of melatonin (MEL) and palmitoylethanolamide (PEA) for the prevention and treatment of immune disease. At first, the intestinal bioaccessibility of PEA and MEL in NF was assessed at 1.6 and 36%, respectively. Then the MEL and PEA ability to modulate the release of immune-inflammatory modulators in the human mast cell line (HMC-1.2) at their bioaccessible concentration was investigated. Our results underline that NF treatment was able to reduce COX-2 mRNA transcription levels (−30% vs. STIM, *p* < 0.0001) in stimulated HMC-1.2 and to contract COX-2 enzymatic activity directly (IC_50_: 152 μg/mL). Additionally, NF showed valuable ability in reducing histamine and β-hexosaminidase release in stimulated HMC-1.2, as well as in decreasing TNF-α and IL-6 mRNA transcription levels and protein production.

## 1 Introduction

Allergic diseases represent a prevalent global health issue that has seen a significant rise in incidence, particularly with the onset of industrialization (1). It is estimated that more than 30% of the global population is affected by one or more allergic-related disorders, especially in Western countries (1). The most common and diffused allergic pathology worldwide include allergies to pollen, house dust mites, animal dander, and obviously food allergens (2). Allergy is an immune-mediated disease, classified as one of the four types of hypersensitivity reactions, formally known as type I (or immediate) hypersensitivity reaction. From the molecular point of view, it is mainly mediated by the interaction of IgE with allergens, leading to a consequent release of mast cell and basophil-derived mediators into the blood system. The allergic reaction could be divided into two different phases, an early step starts from the moment in which the allergen binds to the mast cell with its specific IgE receptors, causing mast cell degranulation, which in turn releases the main molecule mediators responsible for the symptoms typical of immune responses, such as histamines, proteases, proteoglycans, and tumor necrosis factor-alpha (TNF-α) (3). The late phase of the allergic event usually begins within 4–6 h after the allergen exposure and involves the further release of additional inflammatory modulators, including IL-6, IL-1β, and IL-13.

Even though immune events can be accurately diagnosed and effectively treated with conventional drug-based therapy, concerns persist regarding the potential side effects of immunomodulatory drugs and the increasing costs associated with medical treatment (4). Therefore, the identification of innovative and alternative natural sources of bioactive compounds useful for the management and prevention of allergic and allergic-related inflammatory diseases is a worldwide challenge. In this context, several natural molecules have proven a remarkable anti-allergic and immunomodulatory potential. For example, several food-derived compounds such as curcumin (5), resveratrol (6), and quercetin (7) have shown valuable immunoregulatory effects both *in vitro* and in clinical models.

In this sense, other natural compounds, palmitoylethanolamide (PEA) and melatonin (MEL) that are both endogenously produced and could be integrated by external supplementation have also shown a valuable potential in the management of allergic events.

Specifically, PEA is an important endogenous lipid mediator belonging to the fatty acid ethanolamine class (8). It was well reported that PEA could effectively contrast the immune response by downregulating mast-cell degranulation (9, 10). Interestingly, PEA is not only endogenously produced, but it could be introduced also with diet. Specifically, PEA is contained in a wide type of food matrices, including the lipid fraction of egg yolk, peanut oil, some varieties of legumes, such as peas and beans (11), as well as in vegetables including tomatoes and potatoes (12). In this regard, several studies underlined the PEA-based treatment as an alternative remedy for the care of immune-related disorders, including allergic dermatitis (13, 14), attenuating airway allergic symptoms in rats (15), and reducing the symptoms of allergic asthma in humans (16).

MEL (N-acetyl-5-methoxy-tryptamine) is a natural substance largely diffused in the plant world (17). It plays a key role in the regulation of several plant physiological functions, such as plant growth, photosynthesis (18), seed germination (19), and protection against abiotic/biotic stress agents (18). In humans, MEL is a hormone produced mainly in the pineal gland, although its valuable amount is also released from other human body districts, such as the gastrointestinal system, epithelial hair follicles, skin, retina, salivary glands, platelets, and lymphocytes (20, 21). In mammals, MEL was commonly known for its, extensively studied, pivotal role in circadian cycle regulation (22), for which it was used as a functional ingredient in nutraceutical formulation for the management of sleep disorders. Additionally, MEL has proven a general anti-inflammatory potential, reducing the expression levels of the most relevant inflammatory mediators, i.e., metallo-proteinase-9 and -2, TNF-α, cyclooxygenase-2 (Cox-2), nitric oxide synthase, nuclear factor kappa-light-chain-enhancer of activated B cells (NF-kB), interleukin 4 (IL-4), and C-reactive protein, in different *in vitro* and pre-clinical inflammation models (22). This evidence was clinically corroborated by the results of a recent systematic review summarizing the effects of MEL-based treatment on the main inflammatory markers plasma levels recorded by 31 different clinical trials conducted on patients affected by a different type of chronic inflammatory disease (23). They described that the IL-6, IL-8, and TNF-α plasma concentrations were statistically decreased after MEL-based supplementation, without significant side effects showed (23). Directly connected with MEL anti-inflammatory activity, several studies have additionally reported the potential MEL activity for the management of immune reactions. Specifically, Liu and colleagues have described that MEL treatment in mice reduced the inflammation state in the allergic airway mice model (24), these results were in line with those described by others, who reported that MEL treatment reduced total nasal symptom scores and serum ovalbumin-specific IgE, in ovalbumin-induced allergic rhinitis model in rats (25). Moreover, additional studies described the positive effects of MEL supplementation on dermatologic allergic diseases, including atopic dermatitis and chronic spontaneous urticaria (26). As described for PEA, MEL could also be introduced with diet. The main MEL food sources include tomato (32 pg./g), red pepper (93 ng/g), strawberries (12 pg./g), mango (699 pg./g), apples (48 pg./g), oranges (150 pg./g), bananas (378 pg./g), and particularly rice (1,006 pg./g) (27, 28).

Based on these considerations, the present study aimed to evaluate the immunomodulatory activity of an innovative nutraceutical formulation (NF) based on the MEL and PEA combination. At first, to assess the effective NF health-promoting effects, the NF intestinal bioaccessibility was calculated through the application of an *in vitro* digestion protocol. After the evaluation of the bioaccessibility of NF, PEA, and MEL, the NF formulation and the pure compounds MEL and PEA were tested on human mast cells (HMC-1.2). To evaluate NF treatment immune-modulatory and allergic-related inflammation effects on HMC, the histamine and β-hexosaminidase release was evaluated, as well as the expression and release of TNF-α and IL-6. Finally, the ability of NF to reduce at the transcriptional level the expression of COX-2, and their capacity to modulate COX-2 enzymatic activity was further explored.

## 2 Materials and methods

### 2.1 Nutraceutical formulation

The studied NF was based on an innovative combination of Ph. Eur. PEA and MEL (ratio 6,000:1). PEA and MEL doses were 300 mg of PEA and 50 μg of MEL. Ph. Eur. PEA powder was a synthetic compound made up of raw materials of vegetable origin and was purchased from Farmalabor SRL (Canosa di Puglia, Italy). MEL powder was a synthetic compound purchased from Sigma-Aldrich (Milan, Italy). The powders were mixed in the selected ratio to obtain a homogeneous mixture, which was the formulation NF.

### 2.2 Simulated gastro-intestinal digestion of melatonin/PEA formulation

A dose of NF formulation was subjected to successive oral, gastric, and intestinal *in vitro* digestion, following a harmonized procedure reported by the COST action INFOGEST network. Simulated digestion fluids, namely gastric fluid (SGF), salivary fluid (SSF), and intestinal fluid (SIF) were prepared according to our previously published protocol (27). Briefly, 1 g of the formulation was mixed with 3.5 mL of SSF at the temperature of 37°C. Next, 0.5 mL of α-amylase solution (75 U/mL), 25 μL of 0.3 M calcium chloride, and 975 μL of water were added and mixed. A solution of 1 M hydrochloride acid (HCl) was added to reduce the pH of the solution to 7, and the mixture was incubated at 37°C for 2 min in an orbital shaker bath at 200 rpm. Then, for simulating gastric conditions, 7.5 mL SGF, 1.6 mL pepsin solution (2000 U/mL), 5 μL 0.3 M calcium chloride, and 695 μL of water were added and thoroughly mixed. Next, a solution of 1 M HCl was used to modify the pH of the solution to 3, and the mixture was incubated for 120 min at 37°C in an orbital shaker bath at 200 rpm. Afterward, to recreate the intestinal stage, 11 mL SIF, 2.5 mL bile salt solution (65 mg/mL), 5 mL pancreatin solution (100 U/mL of trypsin activity), 1.3 mL of water, and 40 μL of 0.3 M calcium chloride were added. After that, the solution was mixed, and 1 M NaOH was added to modify the pH of the mixture to 7. The solution was incubated at 37°C for 120 min in an orbital shaker bath at 200 rpm. At the end of the incubation, the samples were centrifuged for 10 min at 37°C at 9000 rpm. Finally, the supernatant was collected and diluted until 30% of acetonitrile to block the enzymatic activity (29). Then, the samples were freeze-dried and the powder obtained was subjected to a hydroalcoholic extraction to remove salts and enzymes added during the simulated gastrointestinal digestion before the analysis. Specifically, to the solid residue, a volume of 10 mL methanol was added. The mixture was vortexed for 1 min and placed in an ultrasonic bath (Branson Fisher Scientific 150 E Sonic Dismembrator) for 10 min. Samples were then shaken on an orbital shaker (Sko-DXL, Argolab, Carpi, Italy) at 600 rpm for 10 min and centrifuged at 9000 rpm for 10 min. The supernatants were collected and stored at 4°C protected from the light. The obtained pellets were re-extracted with 10 mL methanol using the same procedure. Finally, the extracted obtained were evaporated to dryness under a light stream of nitrogen, reconstituted in an aqueous solution at 50% methanol at a concentration of 10 mg/mL, and stored at −20°C until analysis. A blank sample of gastro-intestinal digestion was prepared following the same procedure previously described, in the absence of a matrix to digest.

### 2.3 PEA and melatonin quantitative analysis by HPLC-DAD

A Jasco Extrema LC-4000 HPLC system (Jasco Inc., Easton, MD, United States), coupled with an autosampler, a binary solvent pump, a diode-array detector (DAD), and a fluorescence detector (FLD), was used for the analysis. The chromatographic analysis was performed according to the following conditions: elution was performed on a Kinetex^®^ C18 column (250 mm × 4.6 mm, 5 μm; Phenomenex, Torrance, CA, United States). The mobile phases were an ammonium acetate buffer 5 mM containing 0.1% formic acid (A) and acetonitrile (B). The elution gradient was performed under the following conditions: 0–5 min, isocratic on 30% phase B; 5–8 min, linear gradient from 30 to 85% B; 8–10 min, linear gradient from 85 to 95% B; 10–15 min, isocratic with 95% B; 15–20 min, isocratic with 30% B for column reconditioning. The separation parameters were as follows: column temperature was set at 30°C, injection volume was 20 μL, and flow rate was set at 1 mL/min. The quantification of PEA was performed at 205 nm with DAD detection, while the quantification of MEL was performed with an excitation wavelength of 280 nm and an emission wavelength of 310 nm.

### 2.4 HPLC-DAD method validation

#### 2.4.1 Linearity and sensitivity of PEA and MEL HPLC-DAD-FLD analysis

Analytical standards of PEA and MEL were used to develop and validate the HPLC-DAD-FLD method used to evaluate the compound’s concentration in the simulated gastrointestinal products. A mixing stock solution of the two standards was prepared at a concentration of 1,000 ppm using HPLC-grade methanol as solvent. A mixing sub-stock solution at a concentration of 500 ppm for PEA and 50 ppm for MEL was prepared by diluting the stock solution with water to obtain a final composition of water/methanol 50:50 (v/v). Three quality control (QC) working solutions (PEA: 500 ppm, 100 ppm, 50 ppm; MEL: 25 ppm, 5 ppm, 1 ppm), eight different calibration working solutions (0.2, 0.4, 0.8, 1.5, 3.0, 6.0, 12.0, and 25 ppm) for MEL analysis and five calibration working solutions (12.5, 25.0, 50.0, 100.0, and 500 ppm) for PEA were prepared by diluting the mixing working solution (PEA at 500 pm and MEL at 50 ppm) with an aqueous solution at 50% methanol. The calibration working solutions were analyzed by HPLC in triplicate. The calibration curves were constructed by plotting the peak area against the standard concentration to evaluate the linearity of the method. Limits of detection (LODs) and limits of quantification (LOQs) were determined to evaluate the sensitivity of the method. Determination of the signal-to-noise ratio is performed by comparing measured signals from samples with known low concentrations of analyte with those of blank samples and previously described LODs establishing the minimum concentration at which the analyte can be reliably detected as is defined as the lowest detectable concentration of analyst that the analytical system can reliably distinguish from the background level (S (signal of compound)/N (signal of noise)) = 3, while LOQ is defined as the lowest quantifiable concentration of analyst that can be measured with a standard level of confidence, and it is typically calculated using (S/N) = 10. Potential interferences at the retention time of the analytes were demonstrated in the gastro-intestinal blank solution at a concentration of 20 mg/mL and the mixture of two analytes at the lower limit of quantification (LLOQ). The absence of peaks at the target retention times in the blank was verified to evaluate the selectivity.

#### 2.4.2 Accuracy and precision of MEL and PEA HPLC-DAD-FLD analysis

As recommended by the ICH guidelines (30), to validate an analytical method, it is essential to determine the accuracy (estimated by calculating the % bias) and precision (estimated by calculating the % CV, coefficient of variation %) of the developed method (27). Accuracy (% bias) was calculated by intraday and inter-day analysis of calibration standards. Three different concentrations of the two compounds were injected 3 times per day (intra-day) and once for 3 consecutive days (inter-day). Precision (% CV, coefficient of variation %) was determined by an intraday and inter-day analysis of the two compounds at 3 different concentrations. Each analyte was injected 3 times per day (intra-day) and once for 3 consecutive days (inter-day).

#### 2.4.3 Matrix effect of PEA and MEL extraction

In order to evaluate the efficacy of the extraction process from the lyophilized bioaccessible fraction the matrix effect was investigated. Specifically, it was investigated by calculating the ratio of the peak area in the presence of matrix (matrix spiked with PEA and MEL post extraction) to the peak area in the absence of matrix (PEA and MEL in acetonitrile). The matrix was spiked with the analyte in triplicate with 1 μg (low), 5 μg (medium), and 10 μg (high) for MEL, and with 50 μg (low), 250 μg (medium), and 500 μg (high) for PEA. The ratio was calculated as follows (31):


$$\text{Matrix effect}\%=\frac{\mathrm{Peak}\;\mathrm{area}\;\mathrm{in}\;\mathrm{presence}\;\mathrm{of}\;\mathrm{matrix}\;}{\mathrm{Peak}\;\mathrm{area}\;\mathrm{in}\;\mathrm{solvent}}\times 100$$

#### 2.4.4 Recovery of PEA and melatonin extraction

The matrix effect was investigated by calculating the ratio of the peak area in the pre-extraction spiked samples (matrix spiked with PEA and MEL pre-extraction) to the peak area in the post-extraction spiked samples (matrix spiked with PEA and MEL post-extraction). The matrix was spiked with the analyte in triplicate with 1 μg (low), 5 μg (medium), and 10 μg (high) for MEL, and with 50 μg (low), 250 μg (medium) and 500 μg (high) for PEA either before (pre-extraction spiked) or after (post-extraction spiked) extraction. The ratio was calculated as follows:


$$\mathrm{Recovery}\%=\frac{\mathrm{Peak}\;{\mathrm{area}}_{pre-\mathrm{extraction}\;\mathrm{spiked}\;\mathrm{sample}}\;}{{\mathrm{Peak}\;\mathrm{area}}_{\mathrm{post}-\mathrm{extraction}\;\mathrm{spiked}\;\mathrm{sample}}}\times 100$$

### 2.5 Cyclooxygenase 2 *in vitro* inhibitory activity assay

The cyclooxygenase 2 (COX-2) inhibitory activity assays were performed using a Cayman Chemical COX Colorimetric Inhibitor Screening Assay Kit (Cayman Chemical, Ann Arbor, MI, United States). The method is able to evaluate the peroxidase activity of COXs by calorimetrically monitoring the appearance of oxidized N, N,N′, N′-tetramethyl-p-phenylenediamine (TMPD) at 590 nm. Samples were divided into a positive control (100% of COX activity), containing 150 μL of 0.1 M Tris–HCl buffer (pH 8.0), 10 μL of heme, and 10 μL of the enzyme, and 10 μL of sample solution at different concentrations. Samples were incubated at 25°C for 5 min, and then 20 μL of arachidonic acid (AA) solution and 20 μL of a colorimetric substrate solution (TMPD) were added. After 2 min of incubation at 25°C, the absorbance at 590 nm was read (32). The COX-2 inhibitory activities were calculated as follows:


$$\%\mathrm{inhibition}=\left(\frac{\mathrm{Activity}\;\mathrm{of}\;COX2-\mathrm{Activity}\;\mathrm{of}\;COX2}{\mathrm{Activity}\;\mathrm{of}\;COX2}\right)\times 100$$

Results were expressed as IC_50_ (inhibitory concentration), which is the concentration of inhibitor required to inhibit COX activity by 50%.

### 2.6 Cell culture

The human mast cells HMC-1.2 cell line was purchased from Sigma-Aldrich (Milan, Italy) and cultured in Iscove’s Modified Dulbecco’s Medium (IMDM) supplemented with 10% fetal bovine serum (FBS), 2 mmol/L L-glutamin, 100 μg/mL streptomycin, 100 U/mL of penicillin, and 0.08% 1-Thioglycerol (all from Gibco; New York, NY, United States) at 37°C in a humidified incubator under 5% CO_2_.

### 2.7 MTT assay

HMC-1.2 cells were seeded in a 96-multiwell plate (5 × 10^4^/well) and treated with increasing concentrations of PEA (2–32 μM), MEL (10–160 nM), and their combination. After 24 h, 25 μL of MTT (Sigma, Milan, Italy) (5 mg/mL in saline) were added and the plate was incubated for 3 h at 37°C in a humidified incubator under 5% CO_2_. After that, the plate was centrifuged at 300 RCF for 3 min and the supernatants were discarded. The dark blue crystals were lysed with 100 μL of a solution containing 50% (v/v) N, N-dimethylformamide, and 20% (w/v) sodium dodecylsulfate with an adjusted pH of 4.5. The optical density of each well was measured at 620 nm using a Multiskan GO microplate reader (Thermo Fisher Scientific, Waltham, MA, United States).

### 2.8 Measurement of β-hexosaminidase

HMC-1.2 cells were plated in a 24-well plate (2 × 10^5^/well) and pretreated with PEA 16 μM, MEL 80 nM, and NF 16 μM + 80 nM and, 1 h later, stimulated with phorbol myristate acetate (PMA) 50 nM and ionophore 1 μM. After 120 min, 50 μL supernatant was transferred into a 96-well plate and β-hexosamine 1 mM (in citrate buffer 0.05 M, pH 4.5) was added. Similarly, once the 24-well plate was centrifuged, the cell pellet was resuspended with the same solution. Following a 37°C incubation of 90 min, the reaction was stopped by adding 0.1 mM Na_2_CO_3_/NaHCO_3_. The absorbance was measured at 405 nm using the Multiskan GO microplate reader (Thermo Fisher Scientific, Waltham, MA, United States).

### 2.9 Measurement of histamine release

For histamine release quantification, supernatants were assayed by a histamine ELISA kit (Abcam, Cambridge, United Kingdom) following the manufacturer’s instructions.

### 2.10 RNA extraction and quantitative real-time PCR

HMC-1.2 mast cells (5 × 10^4^/well) were pretreated with PEA, MEL, and NF at the same concentrations as the previous experiment for 1 h and then stimulated with PMA 50 nM and ionophore 1 μM. Cells were incubated for 6 h, and then total RNA was extracted using TRI-Reagent (Sigma-Aldrich, Milan, Italy), according to the manufacturer’s instructions. Subsequently, the retro-transcription was performed using iScript Reverse Transcription Supermix (Bio-Rad) and qPCR was carried out in the Bio-Rad CFX384 real-time PCR detection system (Bio-Rad, Milan, Italy) with the following primers:

IL-6: 5′-CGGAGAGGAGACTTCACAGAG-3′; 5′-ATTTCCACGATTTCCCAGAG-3′.

TNF-A: 5′-CAGTAGACAGAAGAGCGTGGT-3′; 5′-AGGCACTCCCCCA AAAGA-3′.

PTGS2: 5′-CTGGCGCTCAGCCATACAG-3′; 5′-CGCACTTATACTGGTCAAATCCC-3′.

Relative gene expression was obtained by normalizing the Ct values against the housekeeping gene ribosomal protein S16 transcript level, using the 2^−ΔCt^ formula.

### 2.11 Cytokine quantification

IL-6 and TNF-α supernatant levels were measured by BioLegend’s LEGENDplex^™^ bead-based immunoassays HU Th Cytokine Panel (12-plex). The analyses were performed according to the manufacturer’s instructions.

### 2.12 Statistics

Statistical analysis was performed using GraphPad Prism software version 9 (San Diego, CA, United States). For the comparison of two groups, a *t*-test was used, and for the comparison of multiple groups, an ANOVA test was used. The data were shown as mean ± SEM. A *p*-value <0.05 was considered statistically significant and was labeled with *; *p*-values <0.01, 0.001, or 0.0001 were labeled with **, ***, or ****, respectively.

## 3 Results

### 3.1 NF bioaccessibility

In order to establish the effectiveness of the proposed formulation, a bioaccessibility study was conducted. Specifically, the NF was subjected to the *in vitro* gastrointestinal digestion protocol, which provides several consecutive steps including, the salivary, gastric, and duodenal phases. The results of the quantitative analysis of the NF duodenal phase are reported in Table 1. Our results indicate that MEL was moderately resistant to the gastrointestinal digestion process, with a calculated duodenal bioaccessibility of 36% vs. undigested matrix. Conversely, PEA was particularly sensible to the gastrointestinal process, leading to a calculated bioaccessible fraction of only 1.59% (Table 1).

**Table 1:** Assessment of duodenal bioaccessibility of MEL and PEA after *in vitro* digestion of an NF day dose.

Compound	Duodenal bioaccessibility
Melatonin	36%
PEA	1.59%

### 3.2 HPLC-DAD-FLD method validation

**Figure 1:**
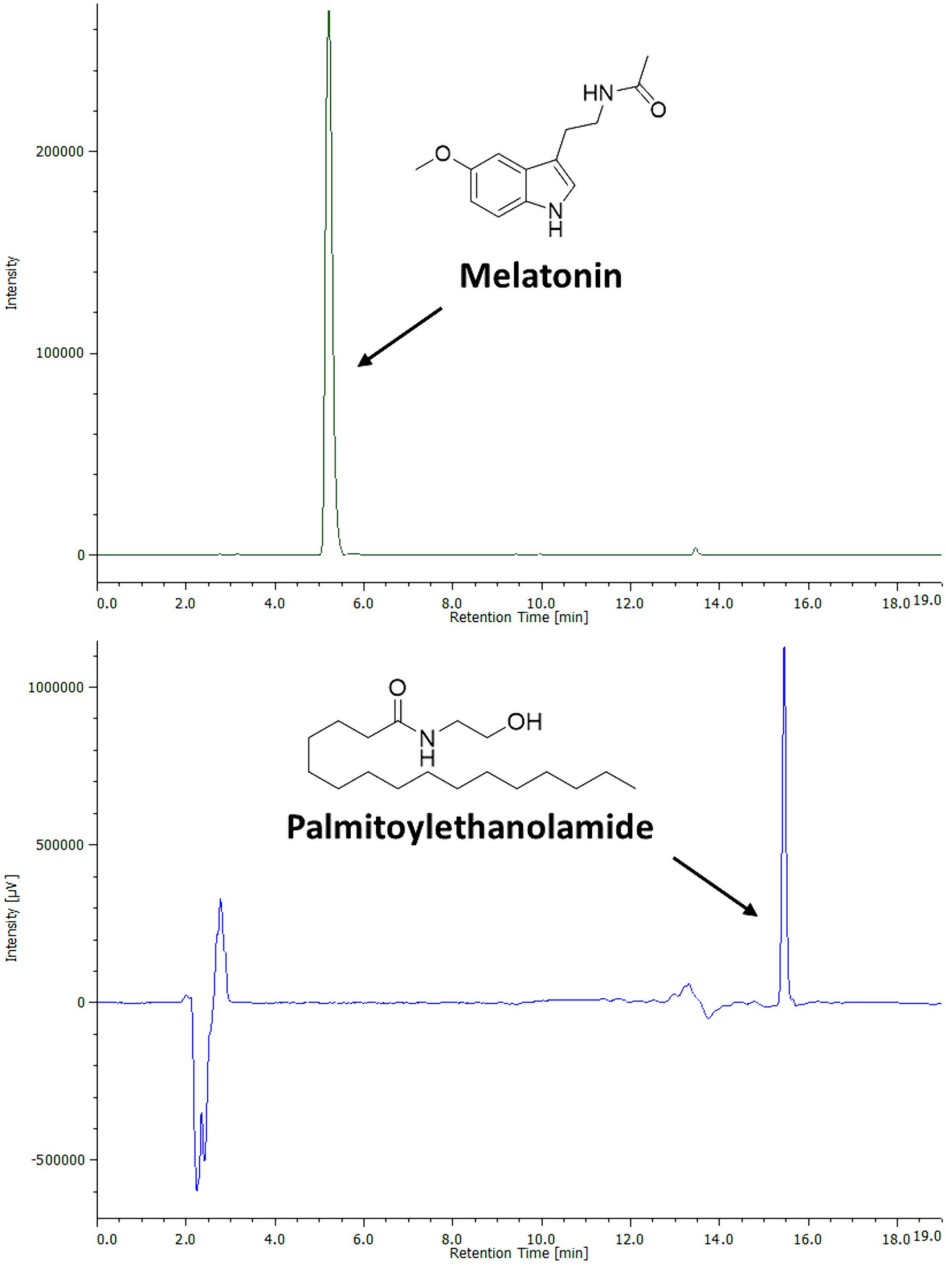
Chromatographic profile and chemical structures of PEA and MEL standards by HPLC-DAD-FLD analysis. PEA (500 ppm) was analyzed by HPLC-DAD at the wavelength of 205 nm. MEL (25 ppm) was analyzed by HPLC-FLD.

In order to identify and quantify simultaneously PEA and MEL in the bioaccessible fraction, a single HPLC-DAD-FLD method was developed. These compounds showed clear chromatographic separation, allowing PEA and MEL simultaneous analysis (Figure 1). The method validation was conducted according to the ICH guidelines (27, 30). Specifically, the linearity studies were conducted by preparing calibration curves on a wide range of calibration points (0.1–1,000 ppm). All determinations were acquired in triplicate, and each analytical standard concentration was plotted versus each peak area, resulting in a linear relation described by a correlation factor *R*^2^ of 0.99. The sensitivity of the analytical method was assessed by determining the LOQ and LOD values for both the studied molecules (Table 2). The obtained data indicate the developed method was 100 times more sensitive in MEL (LOD: 0.1 ppm; LOQ 0.3 ppm) than PEA detection (LOD: 10 ppm; LOQ: 30 ppm). Since both PEA and MEL LOD and LOQ values were largely below the lowest concentrations detected and quantified in all samples analyzed, this analytical method may be considered a reliable protocol for simultaneous MEL and PEA detection and quantification.

**Table 2:** Linearity and sensitivity of PEA and MEL detection.

Compound	Calibration line	*R* ^2^	LOD (ppm)	LOQ (ppm)
Palmitoylethanolamide	*y* = 3 × 10^6^*x* + 32,333	0.99	10	30
Melatonin	*y* = 5 × 10^7^*x* + 28,200	0.99	0.1	0.3

LOQ, limit of quantification; LOD, limit of detection.

Furthermore, intra-day and inter-day accuracy (% bias), and precision (% CV, coefficient of variation %) (33) were also calculated at four different MEL (25, 5, 1, and 0.3 ppm) and PEA (500, 100, 50, and 30 ppm) concentration levels (Table 3). As expected, the higher % CV values were obtained at the lowest concentrations analyzed for both compounds. Specifically, PEA at the lowest concentration tested (30 ppm) showed an intraday and interday % CV of 4.4 and 8.8, respectively. The same trend was also followed by MEL, where the highest % CV values were acquired at 0.3 ppm MEL concentration level (Table 3). In addition, the % bias ranged from 0.1% to −1.8% for the estimation of intra-day PEA accuracy and from −0.6 to −1.7% for the determination of PEA inter-day accuracy. Higher accuracy was found for MEL detection, described by low % bias values, both intraday and inter-day determinations at all the concentration levels tested (Table 3). Both precision and accuracy displayed values lower than 15%, which is considered a limit criterion normally accepted for analytical method validation (27, 28).

**Table 3:** Intra-day and inter-day precision and accuracy of melatonin and PEA detection.

Compound	Concentration (ppm)	Precision (% CV)		Accuracy (% bias)	
		Intraday	Interday	Intraday	Interday
Palmitoylethanolamide	500	1.9	3.3	0.1	−0.6
	100	2.2	3.2	2.4	2.2
	50	4.0	7.1	1.0	0.4
	30	4.4	8.8	−1.8	−1.7
Melatonin	25	0.7	0.2	−0.1	−0.1
	5	0.3	0.3	0.2	−0.2
	1	2.6	1.3	0.3	0.1
	0.3	4.7	4.0	−0.1	−0.4

### 3.3 MEL and PEA recovery and matrix effects in NF duodenal bioaccessible fraction

Due to the complex composition of NF bioaccessible fractions, the assessment of extraction protocol efficacy is highly required. Thus, the recovery (%) and the matrix effect (%) were determined at PEA and MEL different spiked concentrations, 50, 250, and 500 μg for PEA and 1, 5, and 10 μg for MEL (Table 4) in NF bioaccessible fraction. As expected, the higher % matrix effect was calculated at the lowest PEA spiked concentration (50 μg), with a calculated % matrix effect of 5.5%. The same trend was followed also by MEL analysis that led to the most relevant matrix effect of −5.4% at the lowest MEL spiked concentration (1 μg). Regarding the recovery % evaluation, PEA and MEL follow a similar trend. While for PEA detection the highest error was calculated at the maximum PEA spiked concentration (500 μg), the MEL recovery highest error was detected at the minimum melatonin spiked concentration (1 μg) (Table 4). Generally, considering the low values obtained for the % matrix effect and % recovery values close to 100%, the present extraction and analysis methods could be considered as a reproducible and reliable protocol for the simultaneous PEA and MEL quantification by HPLC-DAD-FLD analysis in a complex sample. In particular, % recovery values ranged between 80 and 110%, which are provided by FDA guidelines as values for a good recovery assay (34).

**Table 4:** Recovery (%) and matrix effect (%) of PEA and MEL extraction process.

Compound	Spike (μg)	Recovery (%)	Matrix effect (%)
Palmitoylethanolamide	50	102.3 ± 6.2	5.5 ± 2.1
	250	102.3 ± 3.7	−1.9 ± 2.0
	500	104.8 ± 5.6	0.4 ± 1.0
Melatonin	1	104.2 ± 6.0	−5.4 ± 4.1
	5	98.2 ± 9.1	1.1 ± 1.2
	10	102.1 ± 3.3	3.8 ± 2.3

### 3.4 Cytotoxic effect of PEA, MEL, and NF on HMC human mast cell lines

**Figure 2:**
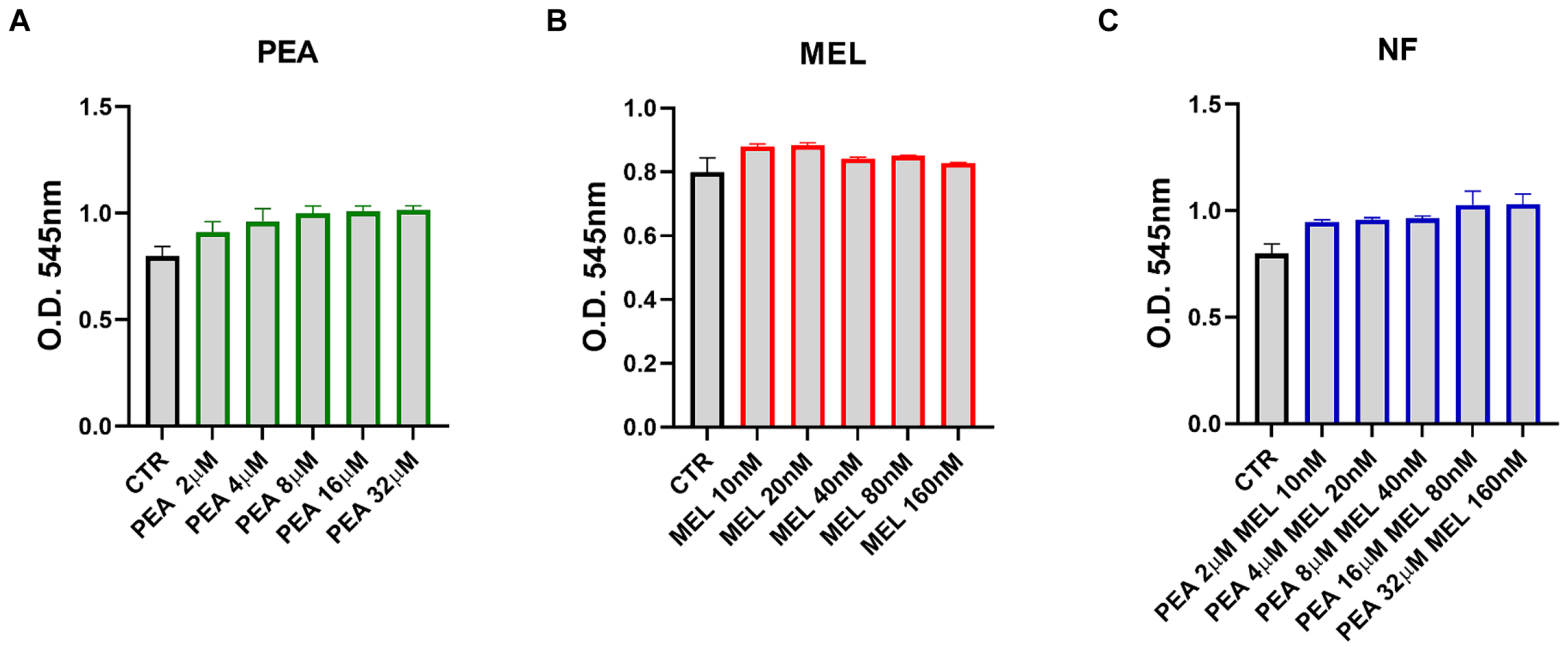
Cytotoxic effect of PEA **(A)**, melatonin **(B)**, and NF **(C)** on HMC-1.2 cells after 72 h evaluated by the MTT assay. Values are expressed as mean ± SEM from three independent experiments.

To evaluate the ability of NF to modulate the activity of mast cells, we used the human mast cell line HMC-1.2 (referred to as HMC). First, we evaluated whether NF, MEL, and PEA single components may exert a cytotoxic effect on HMC cells. Cells were treated with increased concentrations of PEA (2–32 μM), of MEL (10–160 nM), and of NF (mix of PEA with MEL, tested exactly at the same concentration evaluated for every single component) for 72 h and cell viability was evaluated by performing an MTT assay. As shown in Figure 2A, we found that all the PEA concentrations tested were safe and did not impair HMC cell viability. Similarly, MEL (Figure 2B) and NF (Figure 2C) did not affect in any way the cell viability at the same concentrations tested. Thus, according to the results obtained in our bioaccessibility experiments, for the next experiments we used the concentrations of 16 μM and 80 nM for PEA and MEL, respectively.

### 3.5 Pretreatment with NF reduced histamine and β-hexosaminidase release in HMC cells

**Figure 3:**
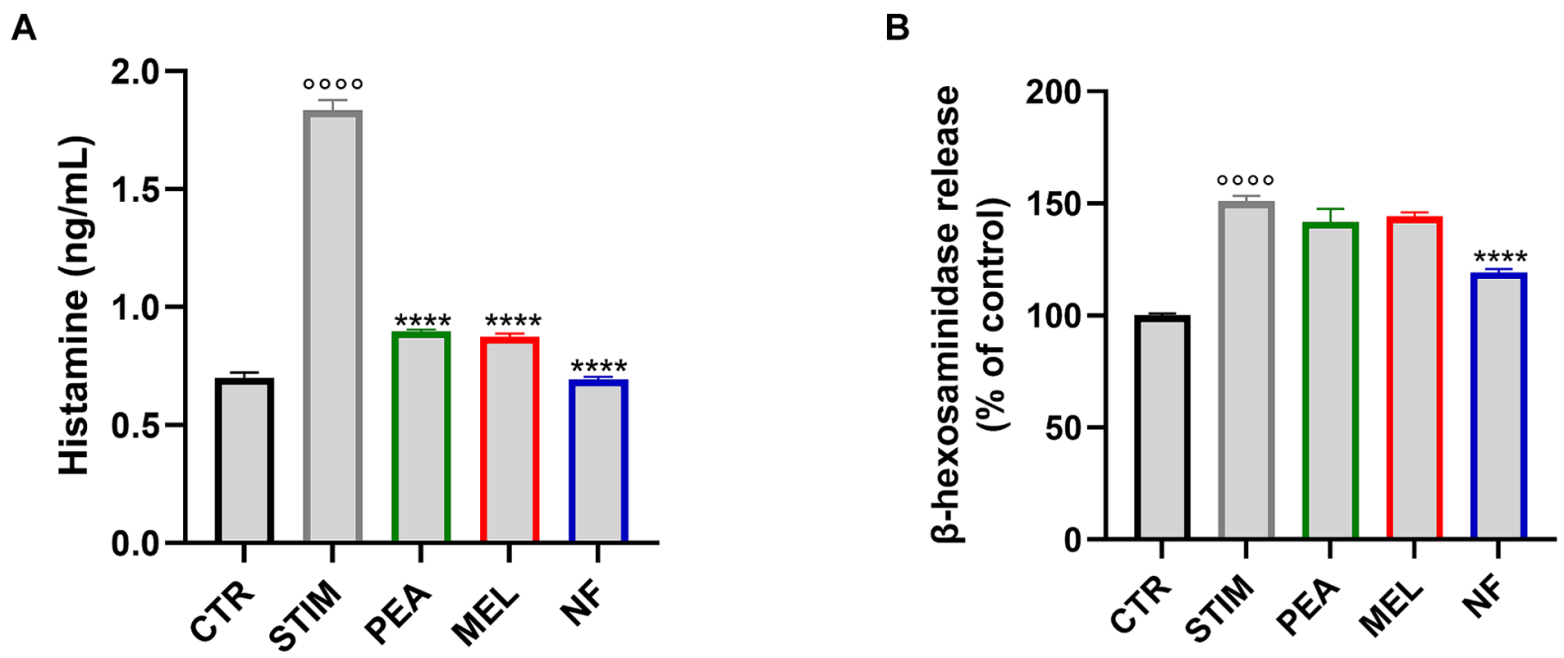
Histamine **(A)** and β-hexosaminidase **(B)** release in HMC-1.2 cells following pretreatment with PEA, MEL, and NF and stimulation with PMA 50 nM and ionophore 1 μM. Values are expressed as mean ± SEM from three independent experiments. °°°°*p* < 0.0001 indicates a significant effect of PMA/Ionophore compared to unstimulated cells (CTR); ****p < 0.0001 indicates significant effect of PEA, MEL, and NF compared to stimulated cells.

To characterize the effect of NF on mast cell activation, we first evaluated the release of histamine and β-hexosaminidase that occur in response to allergens or inflammatory agents, acting as important players in the inflammatory response. Thus, we pretreated HMC cells with NF or PEA and MEL alone for 1 h before stimulation with PMA and Ionophore for 2 h, the most used triggers for mast cell degranulation (referred to as STIM). As shown in Figure 3A, the stimulation with PMA and Ionophore significantly increased the release of histamine. In contrast, pretreatment with NF as well as the single compounds significantly reduced the release of histamine. In line with this result, stimulation with PMA Ionophore induced b-hexosaminidase release by HMC1, which was significantly reduced by NF (Figure 3B). These results highlight the potential synergistic effects of NF in inhibiting the degranulation of mast cells.

### 3.6 NF reduced the mRNA expression and release of pro-inflammatory cytokines in HMC cells

**Figure 4:**
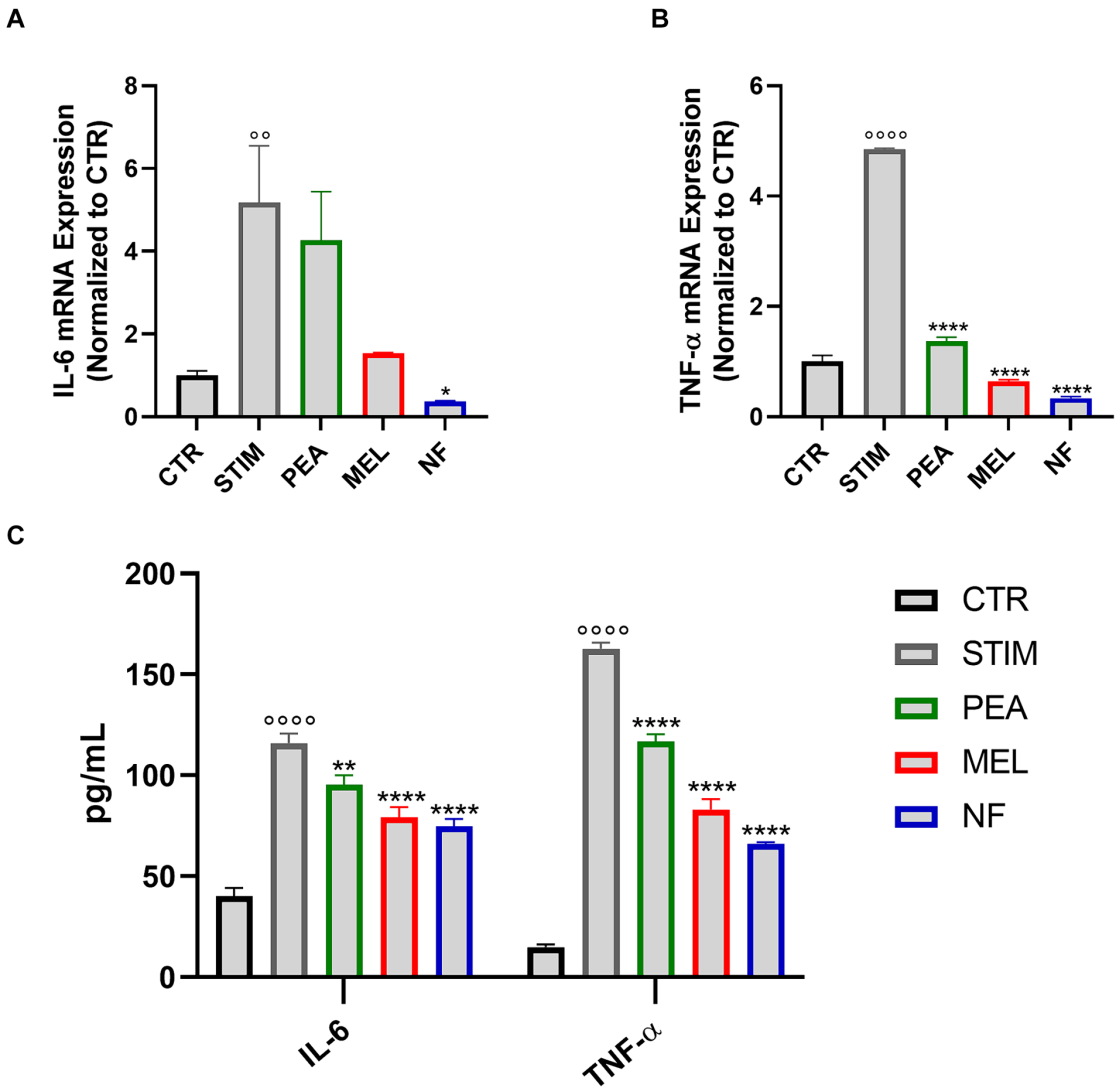
Expression of IL-6 **(A)** and TNF-α **(B)** assessed with qPCR analysis in HMC-1.2 cells pretreated for 1 h with PEA 16 μM, melatonin 80 nM and NF 16 μM + 80 nM and stimulated with PMA 50 nM and ionophore 1 μM for 6 h. **(C)** Levels of IL-6 and TNF-α in supernatants assessed by multiplex assay. Values are expressed as mean ± SEM from three independent experiments. °°°°*p* < 0.0001 indicates a significant effect of PMA/Ionophore compared to unstimulated cells (CTR); *p < 0.05, **p < 0.01, ***p < 0.001, and ****p < 0.0001 indicate a significant effect of PEA, MEL or NF compared to stimulated cells.

Once activated, mast cells are able to release different cytokines that sustain the allergic-related inflammatory response. Therefore, we evaluated by qPCR analysis the RNA expression levels of both TNF-α and IL-6 in NF-treated HMC cells. As expected, the stimulation with PMA and Ionomycin significantly increased the RNA expression levels of the two cytokines tested. Conversely, as shown in Figures 4A,B, the pretreatment with NF, PEA, and MEL significantly reduced the expression of both TNF-α and IL-6. Interestingly, this effect was more pronounced in HMC cells treated with the NF compared to the single compounds. These results were confirmed by the quantification of cytokines in cell culture supernatants of HMC cells, pretreated or not with NF, PEA, and MEL (Figure 4C). In line with the qPCR analysis, we observed a significant reduction of both cytokines in the cultured medium of HMC. However, the pretreatment with NF showed a major reduction compared to the single compounds.

### 3.7 NF reduced the expression and inhibited the activity of the COX-2 enzyme

**Figure 5:**
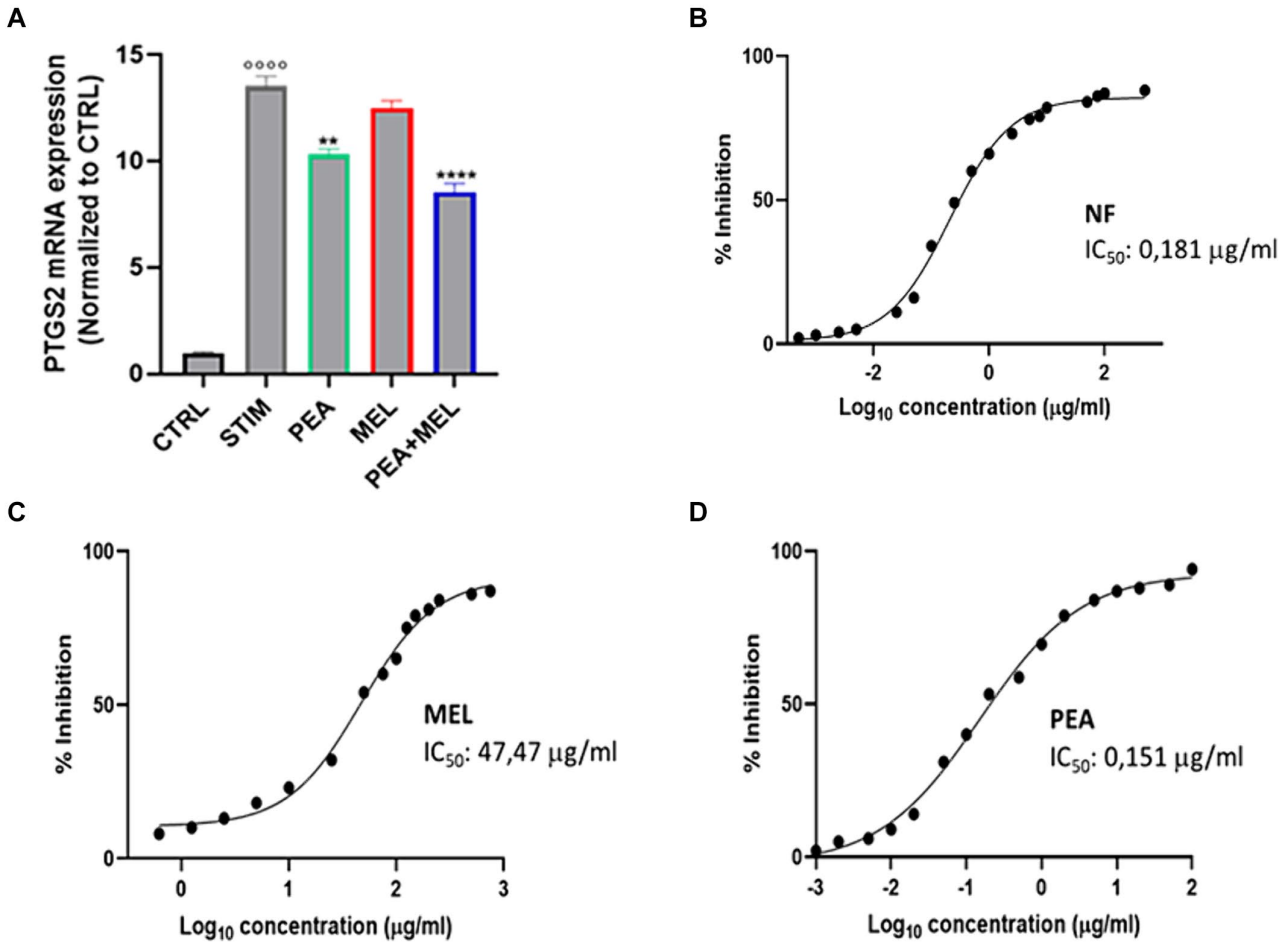
**(A)** Expression of PTGS2 assessed with qPCR analysis in HMC-1.2 cells treated with PEA 16 μM, melatonin 80 nM, and NF 16 μM + 80 nM for 1 h following stimulation with PMA 50 nM and ionophore 1 μM and treatment for 6 h. Inhibition of COX-2 activity (expressed in %) of PEA **(B)** MEL **(C)** and NF **(D)**. Values are expressed as mean ± SEM from three independent experiments. °°°°*p* < 0.0001 indicates a significant effect of PMA/Ionophore compared to unstimulated cells (CTR); **p < 0.01 and ****p < 0.0001 indicate a significant effect of PEA, MEL or NF compared to stimulated cells.

It is well known that type-2 cyclooxygenase enzyme (COX-2) is the inducible form of COX enzyme that sustains the inflammatory process by modulating the function of different immune cells including mast cells (35). Thus, we evaluated the effect of PEA, MEL, and NF on the expression of COX-2 enzyme at transcriptional levels by qPCR analysis. As shown in Figure 5A, stimulation with PMA/Ionophore increased the expression of COX2 whereas pretreatment with PEA, MEL, and NF resulted in a valuable reduction in COX-2 expression vs. STIM. Specifically, the NF was shown more relevant effects on COX-2 transcription rate than its single components, PEA and MEL. Moreover, we also tested the ability of our compounds to directly inhibit the enzymatic activity of COX2. Our results indicate that both MEL and PEA tested separately at concentration levels in the range of their previously described bioaccessible concentrations show a valuable COX-2 inhibitory activity with a calculated IC_50_ of 47.47 μg/mL and of 0.152 μg/mL (Figures 5B,C), for MEL and PEA, respectively. The inhibitory activity of the NF was described by a calculated IC_50_ of 0.135 μg/mL (Figure 5D), with a non-significant difference from the PEA anti-COX2 activity described. Regarding MEL, despite showing a valuable enzymatic inhibitory activity, its IC_50_ of 47.47 μg/mL (204.3 nM) is higher than its calculated bioaccessibility, thus this result has weak health significance. Collectively, these results demonstrated that NF, on the one hand, was able to modulate the expression of COX-2 enzyme at mRNA levels in stimulated HMC-1.2, and, on the other hand, was able to directly contract its enzymatic activity with a calculated IC_50_ of 0.208 μg/mL, a concentration largely below its intestinal bioaccessibility.

## 4 Discussion

Allergic diseases represent a prevalent global health issue with an increasing incidence, particularly in Western countries. Allergic events are characterized by an immune-mediated inflammatory reaction to typically harmless environmental allergens, leading to the proliferation and activation of mast cells, heightened production, and release of IgE in the bloodstream (36). From the molecular point of view, this process was mediated by multistep complex molecular mechanisms that modulate the activity of different immune cells both from the innate and adaptive arms.

Thus, the inhibition or the expression-regulation of such molecular modulators could be considered a useful tool for the management of immune response. In this context, the NF exhibited a valuable anti-allergic activity, acting in both the early and late phases of immune events. Nevertheless, concerning the assessment of nutraceutical formulation’s human health effectiveness, the evaluation of its intestinal bioaccessibility plays a key role. Thus, in the current study, at first, the intestinal bioaccessibility of PEA and MEL was calculated, after the *in vitro* gastrointestinal digestion protocol. Our results indicate that the digestion process drastically reduces the effective active concentration of PEA, with a bioaccessible concentration of only 1.59% concerning the initial concentration, while for MEL a valuable resistance during the digestion process was detected, with a calculated bioaccessibility of 36%. These results, describe a first attempt to define the intestinal bioaccessibility of MEL and PEA in biocomponent nutraceutical formulation. Concerning MEL, scant literature evaluates its bioaccessibility, particularly in its pure form. A single study estimated 80% of MEL bioaccessibility encapsulated in a formulation based on glycosylated egg proteins (37), while another investigation reported that MEL contained in pistachios seeds has shown intestinal bioaccessibility of 21% (38). Interestingly, despite the widespread use of PEA in nutraceutical formulations and its numerous biological activities, there is a surprising lack of scientific evidence addressing this specific topic.

From an analytical point of view, to produce reliable and accurate data about the PEA and MEL intestinal bioaccessibility, these parameters were calculated using an opportunely validated HPLC-DAD-FLD method, which allows their simultaneous accurate and sensitive determination in bioaccessible fraction. According to the analytical validation process conducted, the current method could be considered, accurate, sensitive, reliable, and reproducible, and the extraction process used for the recovery of PEA and MEL from bioaccessible fraction was exhaustive and efficient.

In this context, once the bioaccessible fractions of PEA (15.95 mM) and MEL (215 nM) for a single dose treatment were assessed, the biological assays performed were conducted in a concentration range below such levels, in order to ensure the plausibility of the health-promoting effects investigated. Thus, at first, we evaluated the HMC-1.2 tolerability through an MTT test. Our results indicate that both PEA, MEL, and NF are particularly well-tolerated in the range tested, not showing any toxic effects. Thus to explore the immunomodulatory effects, their activity on the histamine and β-hexosaminidase release from HMC-1.2 stimulated cells was explored. Specifically, these two mediators play a pivotal role in the progression of the initial phase of the allergic reaction, where histamine is responsible for the main symptoms associated with the allergic and immune reaction inducing smooth muscle contraction, heightened vascular permeability, and vasodilation, while β-hexosaminidase is responsible for the progression of inflammatory response. In this sense, β-hexosaminidase contributes to the modification of the extracellular matrix, catalyzing the removal of N-acetylglucosamine (GlcNAc) groups from glycosaminoglycans structural components of the extracellular matrix, facilitating the dispersion and mobilization of inflammatory cells and mediators, enhancing the progression of the allergic-inflammatory response. However, the stronger effect of NF on histamine compared to β-hexosaminidase could be related to the slow release of the latter compared to histamine whose release is faster during mast cell degranulation (39).

Our results highlight that the treatment with both MEL (80 nM) and PEA (16 μM) was able to decrease the level of histamine release rate by 55% vs. STIM (*p* < 0.0001), while the treatment with NF led to a more evident reduction of histamine release (65% vs. STIM, *p* < 0.0001) than the single component treatment. Conversely, the release of β-hexosaminidase follows a completely different trend, while the single MEL and PEA treatment led to weak effects (with no significative difference vs. STIM), but their combination shows a valuable reduction in terms of β-hexosaminidase release. In this regard, the ability of MEL to reduce β-hexosaminidase release in a dose-dependent manner (over the concentration of 0.2–1 mM) in rat basophilic leukemia (RBL-2H3) cells has already been reported (40). On the same cellular model was additionally studied the PEA ability to reduce the histamine and β-hexosaminidase in dose dose-dependent manner. Particularly, Petrosino and colleagues stated that PEA treatment at the concentration range of 0.1–10 mM strongly reduced the release of β-hexosaminidase and histamine in stimulated RBL-2H3 cell lines (13). Additionally, other authors have described the same PEA treatment effects in canine mast cells isolated from skin biopsies, at the concentration of 30 mM could simultaneously reduce the histamine and TNF-α release by 60 and 40%, respectively (41).

Molecularly, these effects could be explained considering the two different molecular pathways separately activated by PEA and MEL. It was well reported that PEA modulates the activity of mast cells by a specific interaction with the cannabinoid receptor, the CB2 receptor (9). Regarding the MEL activity on mast cells, it was well described that MEL could exert a direct effect on HMC cells through the interaction with MEL receptors 1 and 2 (MT1 and MT2), two G-protein–coupled receptors, expressed in human mast cells. Specifically, MEL through their activation could lead to the inhibition of NF-κB activation, which in turn could downregulate MC degranulation, proliferation, and differentiation, resulting potentially in a reduction of histamine release (42). Thus, in light of the difference, the results obtained could be explained as considered a synergic activation of both molecular via described. Interestingly, considering the role of mast cell activation in the pathogenesis of coeliac disease and inflammatory bowel diseases (IBD) (43, 44), the PEA and MEL ability to modulate the mast cell degranulation could be considered as a potential alternative treatment for intestinal-immune-based disorders.

Next, to investigate the NF potential immunomodulatory activity in the tardive phase of the immune response process, its ability to regulate both at the transcriptional level and the production of IL-6 and TNF-a was investigated. The NF treatment leads to a valuable reduction in mRNA expression levels for both IL-6 and TNF-α, and reasonably leads to decreased synthesis of these cytokines and interleukins. These data were supported by previously published findings, reporting that PEA through interaction with PPARγ in RBL-2H3 and rat mast cells, leads to a reduction of cytokine release, including TNF-α in both models (9, 45). Regarding MEL, other evidence described that the pre-treatment with MEL (100 nM) in stimulated mast cells, significantly reduced the levels of TNF-α and IL-6 via inhibition of IKK/NF-kB (46). Based on such consideration, it could be hypothesized the NF’s greater effect in reducing IL-6 and TNF-α than its single components, could be related to a synergic combination of both the mechanism of action mentioned above, the PEA component acting via PPARγ while the MEL component via IKK/NF-kB.

Finally, we moved to investigate the NF’s ability to transcriptionally modulate the expression of COX-2 in stimulated HMC-1.2 cell lines. Our results indicate that NF, has a powerful activity in reducing at the transcriptional level the COX-2 expression (−30% vs. STIM, *p* < 0.0001), while no statistically significant results were obtained after the monocomponent treatment, suggesting a potential synergic effect. These results were in line with other published data where PEA demonstrated to downregulate the COX-2 mRNA expression level (47).

Once established the NF efficacy in reducing the COX-2 transcription rate, we investigated whether NF could also exert a direct inhibitory effect on the COX-2 enzymatic activity. Our results did not confirm the same trend shown for the data obtained at the transcription level. Above all, NF has shown a comparable COX-2 inhibitory activity to PEA single-component treatment, with a similar calculated IC_50_ value (0.151 μg/mL for PEA and 0.198 μg/mL for NF)_._ Not surprisingly, these could be related principally to the fact that PEA has shown a higher inhibitory potential toward COX-2 enzymatic activity than MEL (IC_50_ 47.47 μg/mL), and, in addition, represents the main functional ingredient in NF. These data agreed with literature data, which reported that PEA has shown a valuable direct inhibitory COX-2 enzymatic activity, with a reported IC_50_ value of 0.57 mM (48), as well as for MEL was confirmed the calculated inhibitory activity by the results of a previously published study where was reported an IC_50_ of 60 μg/mL (49).

## 5 Conclusion

This study represents the first attempt to evaluate a potential synergic immunomodulatory activity of innovative nutraceutical formulation based on the combination of PEA and MEL. Our results underline that both these components, at concentration levels lower than their intestinal bioaccessible calculated fractions, can positively modulate the mast cell degranulation, and consequently reduce the liberation of the main immune-inflammatory molecular modulators, i.e., TNF-α, IL-6, and COX-2 in treated HMC-1.2. In conclusion, this formulation could be considered as a potential alternative remedy for the treatment of allergic or immune-inflammatory diseases at different body districts, including at the intestinal level. Nevertheless, further studies are required to establish their bioavailability and to clarify the specific molecular pathways involved in the collected results.

## Data Availability

The original contributions presented in the study are included in the article/supplementary material, further inquiries can be directed to the corresponding author.
